# Identification of early biomarkers of transcriptomics in alveolar macrophage for the prognosis of intubated ARDS patients

**DOI:** 10.1186/s12890-022-02130-8

**Published:** 2022-09-02

**Authors:** Songchang Shi, Shuo Wei, Xiaobin Pan, Lihui Zhang, Shujuan Zhang, Xincai Wang, Songjing Shi, Wei Lin

**Affiliations:** 1 Department of Critical Care Medicine, Shengli Clinical Medical College of Fujian Medical University, Fujian Provincial Hospital South Branch, Fujian Provincial Hospital, Fuzhou, 350001 People’s Republic of China; 2 Department of Infectious Disease, Shengli Clinical Medical College of Fujian Medical University, Fujian Provincial Hospital, Fuzhou, 350001 People’s Republic of China; 3 Department of Endocrinology, Shengli Clinical Medical College of Fujian Medical University, Fujian Provincial Hospital, Fuzhou, 350001 People’s Republic of China; 4 Department of Critical Care Medicine, Shengli Clinical Medical College of Fujian Medical University, Fujian Provincial Hospital, Fuzhou, 350001 People’s Republic of China

**Keywords:** Biomarkers, Transcriptomics, Prognosis, ARDS, Alveolar macrophage

## Abstract

**Background:**

Currently, the rate of morbidity and mortality in acute respiratory distress syndrome (ARDS) remains high. One of the potential reasons for the poor and ineffective therapies is the lack of early and credible indicator of risk prediction that would help specific treatment of severely affected ARDS patients. Nevertheless, assessment of the clinical outcomes with transcriptomics of ARDS by alveolar macrophage has not been performed.

**Methods:**

The expression data GSE116560 was obtained from the Gene Expression Omnibus databases (GEO) in NCBI. This dataset consists of 68 BAL samples from 35 subjects that were collected within 48 h of ARDS. Differentially expressed genes (DEGs) of different outcomes were analyzed using R software. The top 10 DEGs that were up- or down-regulated were analyzed using receiver operating characteristic (ROC) analysis. Kaplan–Meier survival analysis within two categories according to cut-off and the value of prediction of the clinical outcomes via DEGs was verified. GO enrichment, KEGG pathway analysis, and protein–protein interaction were also used for functional annotation of key genes.

**Results:**

24,526 genes were obtained, including 235 up-regulated and 292 down-regulated DEGs. The gene ADORA3 was chosen as the most obvious value to predict the outcome according to the ROC and survival analysis. For functional annotation, ADORA3 was significantly augmented in sphingolipid signaling pathway, cGMP-PKG signaling pathway, and neuroactive ligand-receptor interaction. Four genes (ADORA3, GNB1, NTS, and RHO), with 4 nodes and 6 edges, had the highest score in these clusters in the protein–protein interaction network.

**Conclusions:**

Our results show that the prognostic prediction of early biomarkers of transcriptomics as identified in alveolar macrophage in ARDS can be extended for mechanically ventilated critically ill patients. In the long term, generalizing the concept of biomarkers of transcriptomics in alveolar macrophage could add to improving precision-based strategies in the ICU patients and may also lead to identifying improved strategy for critically ill patients.

**Supplementary Information:**

The online version contains supplementary material available at 10.1186/s12890-022-02130-8.

## Introduction

Acute respiratory distress syndrome (ARDS), accompanied by increased pulmonary vascular permeability, and loss of aerated lung tissue, is an acute lung injury [1]. A series of interventions have been proposed for ARDS, such as lower tidal volumes [2], higher positive end-expiratory pressure (PEEP) [3], prone positioning [4], and extracorporeal membrane oxygenation [5]. It was found by a recent international, multicenter observational cohort study that morbidity and mortality of ARDS were still high [6]. The prevalence period of ARDS among ICUs in 50 countries was 10.4% of ICU admissions [6]. Overall, up to 40% of patients with ARDS died in the hospital [6].

One of the potential reasons for the lack of effective therapies is the absence of early, credible indicator for risk prediction, which would help in the precise treatment of acute ARDS [7, 8]. It was shown that 1/3 of the genes in blood leukocytes were differentially expressed between sub-phenotypes of ARDS, supporting the biological heterogeneity of patients [9]. These biological sub-phenotypes are suggested to provide prediction for a precision-based therapeutic strategy [10].

Recent studies have suggested that transcriptomic analyses using whole-blood leukocyte RNA might not accurately reflect all the lung processes of ARDS [11]. Other findings suggest that alveolar macrophages (AMs) may contribute towards the inflammation and injury in ARDS [12]. Nevertheless, assessment of the clinical outcomes with ARDS by AM has not been performed [12].

In this study, we examined the transcriptome of AMs isolated from patients on the first day after the onset of ARDS. Our study attempted to performed the assessment of the clinical outcomes with transcriptomics of ARDS by alveolar macrophage (AM).

## Methods

### Study population and design

**Fig. 1:**
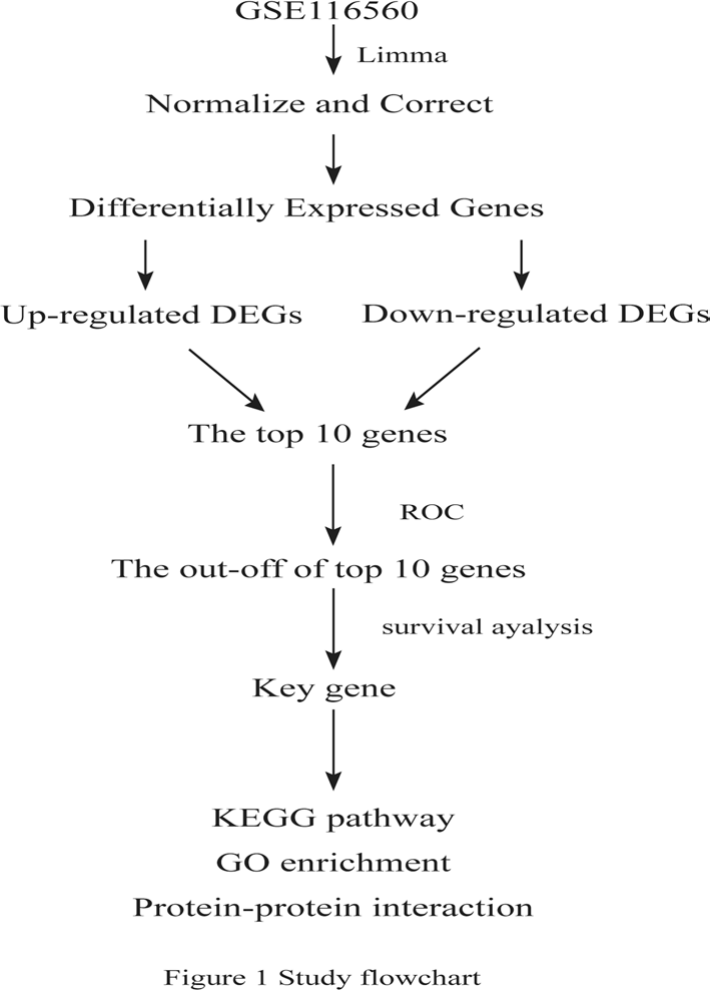
Study flowchart

The data was obtained from the Gene Expression Omnibus databases (GEO) in NCBI, a public functional genomics data repository. The expression data (https://www.ncbi.nlm.nih.gov/geo/; GSE116560) was based on the Illumina GPL6883 platform (Illumina Human Ref-8 v3.0 expression BeadChip), which was submitted by Charib SA et al. This data performed unbiased genome-wide transcriptional profiling of AMs purified from bronchoalveolar lavage fluid collected from patients with ARDS. This dataset [12] consists of 68 samples from 35 subjects that occurred within 48 h of ARDS, whose BALF (BAL fluid) were taken at Day 1, Day 4 and Day 8. The average age was 45, comprising 22 men and 13 women. The factors of the ARDS were trauma (19), sepsis (17), pneumonia (9), and other (4). Participants who extubated were not selected for bronchoscopy on Day 4 or Day 8. Twenty patients were successfully extubated within 28 days. Fifteen patients were unsuccessfully extubated at 28 days, including five patients who died. At present, 28 days have been observed as an important time point for prognosis in many studies on ARDS [13–15]. The patients who were released from mechanical ventilation within 28 days were defined as the "good" group, those who died or were still dependent on mechanical ventilation at Day 28 were defined as the "poor" group. The data was prepared, processed and analyzed by the software R (version 3.6.3). The flowchart of this study is shown in Fig. 1. The procedures followed were in accordance with the ethical standards of the Responsible Committee on Human Experimentation and with the Helsinki Declaration.

### Identification of differentially expressed genes

The data was normalized and corrected by the package limma of R software [16]. Differentially expressed genes (DEGs) of different outcomes were screened with criteria |log2fold change (FC)|> 0 and *p* value < 0.05 for statistically significant difference. Consequently, the DEGs were divided into two groups namely, up-regulated DEGs and down-regulated DEGs.

### ROC analysis of top 10 genes DEGs

The top 10 genes with up- and down-regulated DEGs were analyzed by receiver operating characteristic (ROC) analysis. Areas under the curve (AUC) and cut-off were determined to evaluate the predicted value and observation point of these top 10 genes using the pROC package of R software [17]. The best observation points of cut-off were obtained according to the Yoden Index.

### Survival analysis of top 10 DEGs

According to the best observation point obtained in ROC, the genes in DEGs were divided into two categories based on expression: (1) greater than the observation point and (2) less than the observation point. Top 10 up-regulated and down-regulated genes were then analyzed with the package of survival as part of R software. Key genes related to prognosis were subsequently screened out based on the results of cox regression of survival analysis.

### Functional annotation of key genes

Key genes were performed by Gene Ontology (GO) [18, 19] enrichment and Kyoto Encyclopedia of Genes and Genomes (KEGG) [20] pathway analysis to investigate the functional annotation. Proteins encoded by genes were associated with cell functions. The information of protein–protein interaction (PPI) was performed with the help of the Search Tool for the Retrieval of Interacting Genes/Proteins (STRING) database [21]. The database of STRING was used to analyze the interaction between DEGs and key selected genes. The software of Cytoscape and its plug-in Multicontrast Delayed Enhancement (MCODE) were used to visualize and select key genes belonging to the PPI network [22, 23]. The cut-off criteria was used with degree = 2, node score = 0.2, k-core = 2, and maximum depth = 100.

## Results

### Identification of DEGs

**Fig. 2:**
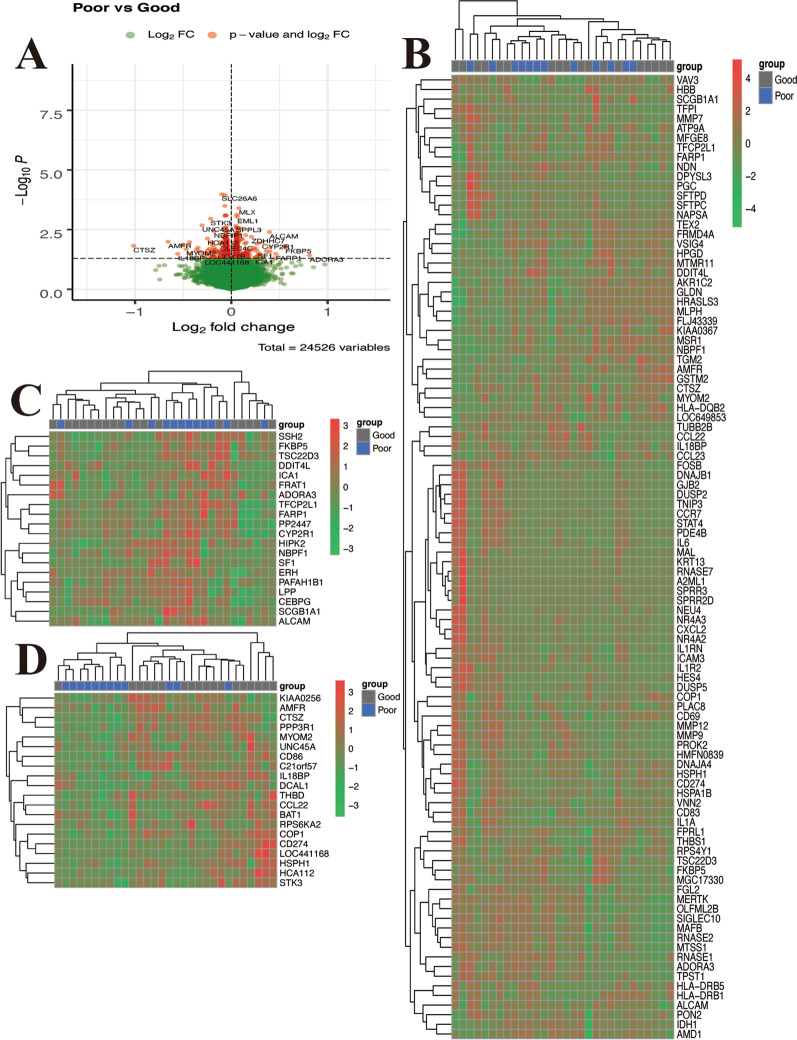
Identification of DEGs

24,526 genes were screened from the GSE116560 dataset, including 235 up-regulated and 292 down-regulated DEGs. It was shown that some of the transcriptional programs of AM were different between the patients released from mechanical ventilation within 28 days and those who died or were still dependent on mechanical ventilation at Day 28.The volcano map of all DEGs is shown in Fig. 2A. The heat map of top 100 DEGs shows top 20 up-regulated genes, and top 20 down-regulated genes, Fig. 2B–D.

### Identification of the top 10 DEGs with outcome

**Fig. 3:**
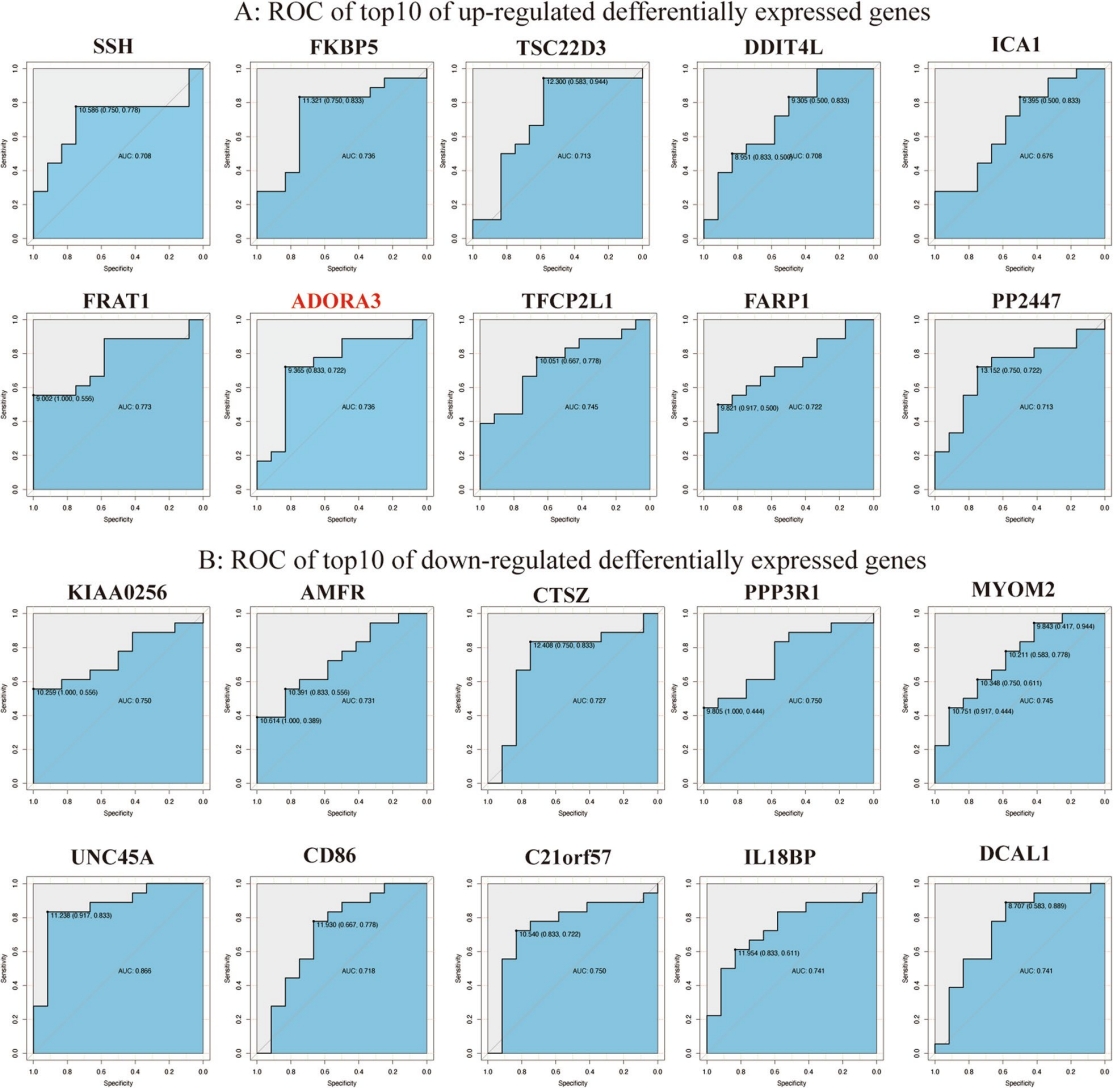
Identification of the top 10 DEGs with outcome

The top 10 up- and down-regulated DEGs were identified based on ROC analysis. The group of genes identified showed the best outcome with the setting AUC > 0.7, except for ICA1 (Fig. 3). The AUC for the gene FRAT1 was 0.773, with a significant change of gene expression as up-regulated DEGs (Fig. 3A). The AUC for the gene UNC45A was 0.866, with the most significant change of gene expression as down-regulated DEGs (Fig. 3B). The best observed concentration of cut-off to predict was chosen after ROC analysis.

### Identification the key genes via survival analysis

**Fig. 4:**
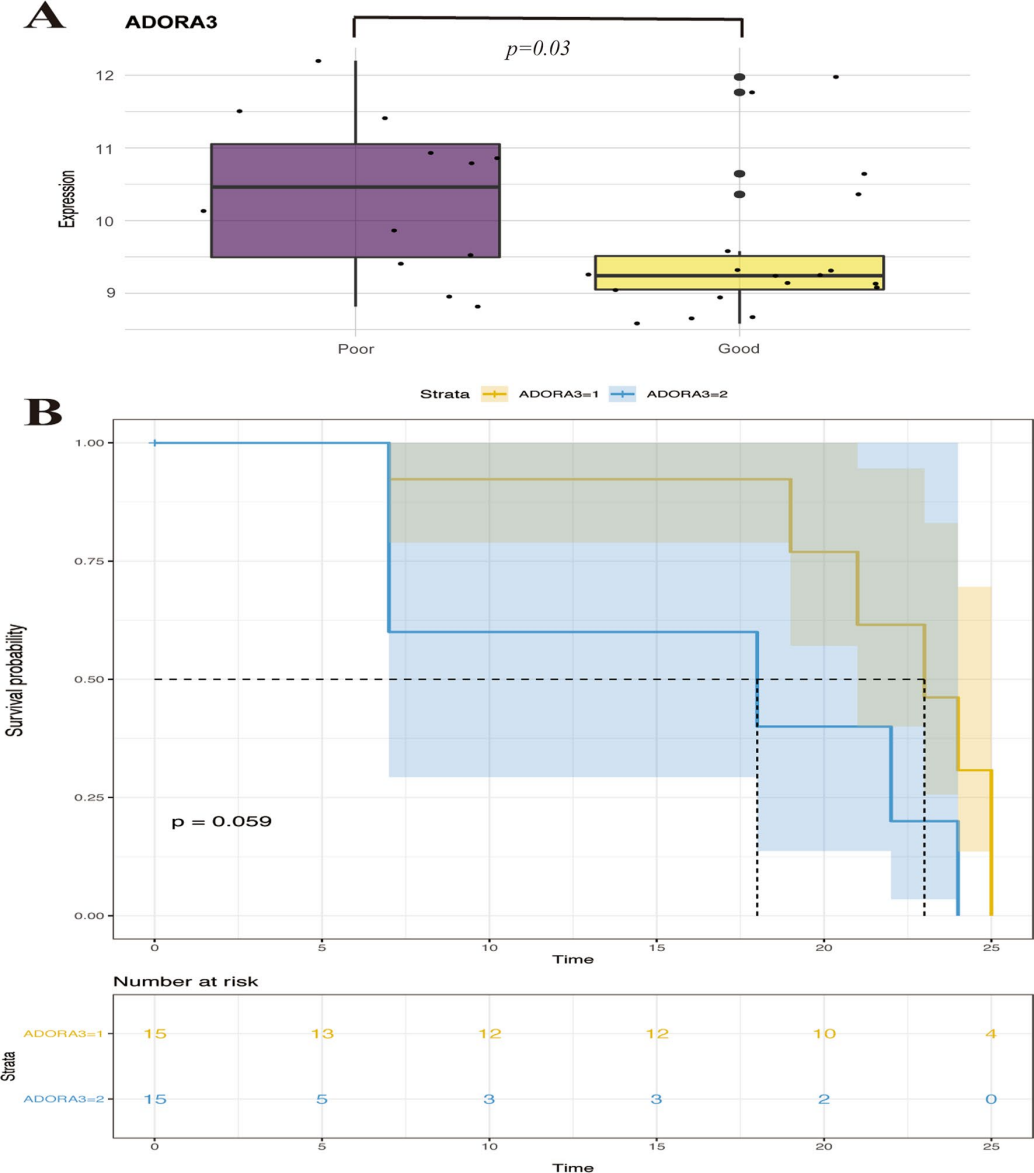
Identification the key genes via survival analysis

Kaplan–Meier survival analysis using two categories based on the cut-off was performed and the value of prediction of the clinical outcomes via DEGs was verified (Table 1). The gene ADORA3 showed a significant change with this outcome (Fig. 4A and Additional file 1). This was chosen as the most appropriate value to predict the outcome according to the survival analysis (Fig. 4B), *p* = 0.059 (< 0.10).

**Table 1:** Survival analysis of the top 10 up-regulated and down-regulated genes

	Genes	OR	Lower 95%	Upper 95%	*p* value
Top 10 of the up-regulated DEGs	SSH2	0.516	0.166	1.603	0.252
	FKBP5	1.102	0.313	3.882	0.879
	TSC22D3	0.368	0.048	2.827	0.337
	DDIT4L (cut-off = 9.305)	1.862	0.487	7.116	0.363
	DDIT4L (cut-off = 8.951)	0.692	0.269	1.780	0.445
	ICA1	0.757	0.217	2.636	0.662
	FRAT1	0.512	0.194	1.347	0.175
	ADORA3	2.285	0.926	8.614	0.068*
	TFCP2L1	0.918	0.302	2.794	0.881
	FARP1	0.607	0.235	1.566	0.301
	PP2447	0.757	0.268	2.141	0.600
Top 10 of the down-regulated DEGs	KIAA0256	0.614	0.240	1.571	0.309
	AMFR (cut-off = 10.391)	1.120	0.440	2.851	0.812
	AMFR (cut-off = 10.614)	0.760	0.293	1.968	0.571
	CTSZ	0.907	0.258	3.195	0.879
	PPP3R1	0.564	0.217	1.465	0.239
	MYOM2 (cut-off = 9.843)	2.715	0.354	20.840	0.337
	MYOM2 (cut-off = 10.211)	0.877	0.287	2.678	0.818
	MYOM2 (cut-off = 10.348)	0.883	0.337	2.314	0.800
	MYOM2 (cut-off = 10.751)	0.728	0.286	1.852	0.505
	UNC45A	1.152	0.332	4.004	0.824
	CD86	0.414	0.119	1.444	0.166
	C21orf57	0.719	0.253	2.044	0.535
	IL18BP	0.786	0.302	2.047	0.622
	DCAL1	0.632	0.141	2.842	0.550

*DEG* differentially expressed genes

*p < 0.10

### Functional annotation of gene ADORA3

**Fig. 5:**
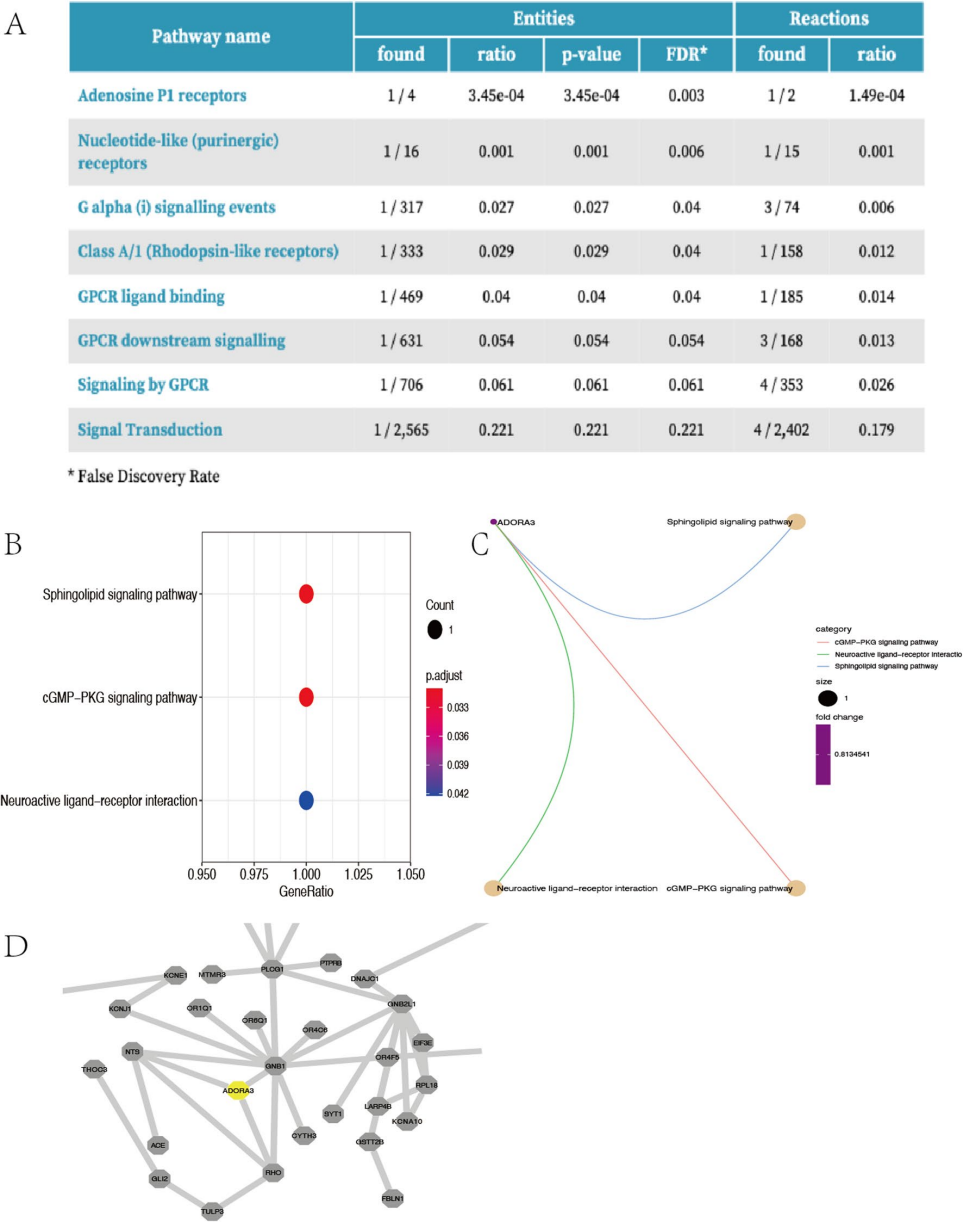
Functional annotation of gene ADORA3

The key gene ADORA3 was further evaluated by GO categories and KEGG pathways. Results of GO [24, 25] analysis found ADORA3 significantly enriched in pathways, such as adenosine P1 receptors, nucleotide-like (purinergic) receptors, G alpha (i) signaling events, GPCR ligand binding, GPCR downstream signaling, signaling by GPCR, and signal transduction (Fig. 5A). The KEGG pathway results revealed that ADORA3 was significantly augmented in sphingolipid signaling pathway, cGMP-PKG signaling pathway, and neuroactive ligand-receptor interaction (Fig. 5B, C).

### Protein–protein interaction network

The PPI network of 237 up-regulated DEGs from ARDS patients identified using STRING analysis contained 123 nodes and 146 edges. A total of 6 clusters were generated in MCODE. 4 genes were selected as the most important clusters by the scores evaluated in MCODE. MCODE 1, consisting of four genes (ADORA3, GNB1, NTS, and RHO), with 4 nodes and 6 edges, had the highest score in these clusters (Fig. 5D).

It was shown that transcriptional programs of AM were different between the patients released from mechanical ventilation within 28 days and those who died or were still dependent on mechanical ventilation at Day 28. Among them, ADORA3 might be a key gene.

## Discussion

This study investigated the prognostic and predictive enrichment of ARDS sub-phenotypes of intubated patients. This is the first study to find the different prognosis between patients with significantly high or low expression of gene ADORA3 in ARDS. In the predictive model of our survival analysis, it forecasted that different expression levels of ARDOA3 at above or below the cut-off, the incidence of extubating were different at 5, 10, 15, 20, and 25 days. Our study revealed that ADORA3 and its related pathways might be associated with 28-day outcomes. As we know, ADORA3 might play an important part in the resolution and initiation of ARDS.

ADROA3 may affect the physiological functions of cells and organs by participating in signal transduction in several signal pathways. Purinergic receptors family includes the GPCR P2Y purinergic receptors and adenosine P1 receptors [26]. Purinergic receptors are involved in cellular functions such as vascular reactivity, apoptosis and cytokine secretion [27]. P1 receptors are purinergic receptors such as G-protein coupled receptors with their endogenous ligand adenosine. There are four adenosine receptors in humans, with distinct functions. They might play important roles in the heart, brain, and might be involved in inflammation and immune responses [28]. The classical mechanism is inhibition of the cAMP dependent pathway via inhibition of adenylate cyclase [29]. Decrease in production of cAMP from ATP leads to decreased activity of cAMP-dependent protein kinases [29].

Putten et al. found that ADORA3 and its pathway induced proinflammatory cytokines [30]. Another research concluded the protective effect of preventing immune mediated damage and excessive immune response via ADORA3 activation [31]. Lung fibroblasts are promoted into myofibroblasts by adenosine receptors. This suggests a potential involvement of ADORA3 in the processes of fibrotic lung disease [32]. Until now, studies on ADORA3 and ARDS pathogenesis is insufficient, and the mechanisms remain to be investigated further.

Some researchers have found the role of ADORA3 in heart. The signaling of ADORA3 protects cardiomyocytes against the damage of ischemia, as well as protects it from energy depletion and contractile dysfunction [33–37]. Moreover, activation of ADORA3 might induce myocardial cells apoptosis [38]. Del et al. found that mRNA expression of ADORA3 in left ventricle of failing minipig heart was higher than in hearts of control healthy minipig [39]. In addition, ADORA3 is overexpressed in inflammation and up-regulated in peripheral blood mononuclear cells of autoimmune diseases. Activation of ADORA3 resulting in downregulation of nuclear factor kappa B (NF-kB) and tumor necrosis factor-α (TNFα) [40], leads to the inhibition of inflammatory cytokines [41].

The package of limma was used from Bioconductor to correct for multiple testing issue. Through the analysis of differential genes by ROC analysis, survival analysis, functional annotation, and PPI network. We narrowed the scope of target genes. The top10 up-regulated and top 10 down-regulated genes had been analyzed by survival analysis for all.

In the survival analysis of ADORA3, it was found that the two groups of survival analysis curves were far apart within the first 3 weeks, and gradually approached closer to each other in the last week. It might be considered that ADORA3 may be useful in the early stages of ARDS to predict mortality, but not in the later stages of the disease. Furthermore, we will stratify the disease course and expand the sample size in the further study.

This datasets we used were obtained from a public database. With this datasets, Morrel et al. [12] identified genes between groups at each time point using linear models, while temporal expression analysis was performed using the STEM. Zhao et al. [39] analyzed that GSE116560 was only one of the datasets, which was based on different platform. In our study, we narrowed the scope by screening differential genes and ROC, and finally performing survival analysis on related genes with a distinctive combination of time and outcome.

Although this study is promising and opens up a new perspective on the impact of ADORA3 and its signaling on the outcome in ARDS, the potential limitations of our research should also be contemplated. First, the dataset was obtained from a public database. All results were obtained using bioinformatics. Future studies are currently being designed for examining the exact roles of ADORA3 and its signaling in ARDS.. Second, because of the limited sample size, studies on a larger population are required to better define the role of ADORA3 in ARDS.

In conclusion, our results show that the prognostic prediction of early biomarkers of transcriptomics as identified in alveolar macrophage in ARDS can be applied to mechanically ventilated critically ill patients. In the long term, generalizing the concept of biomarkers of transcriptomics in alveolar macrophage could add to improving precision-based strategies in the ICU population and lead to identifying treatable therapy for all critically ill.

## Supplementary Information

**Additional file 1. Supplementary 1** Different expression of the key genes with the outcome.

## Data Availability

The datasets analyzed during the current study are publicly available in the [Gene Expression Omnibus databased] repository/database, [https://www.ncbi.nlm.nih.gov/geo/; GSE116560].
