# Supplementary figures and images for: Identification of early biomarkers of transcriptomics in alveolar macrophage for the prognosis of intubated ARDS patients
Source: BMC Pulm Med. 2022 Sep 2;22:334. doi: 10.1186/s12890-022-02130-8 (PMC9440545; doi:10.1186/s12890-022-02130-8)

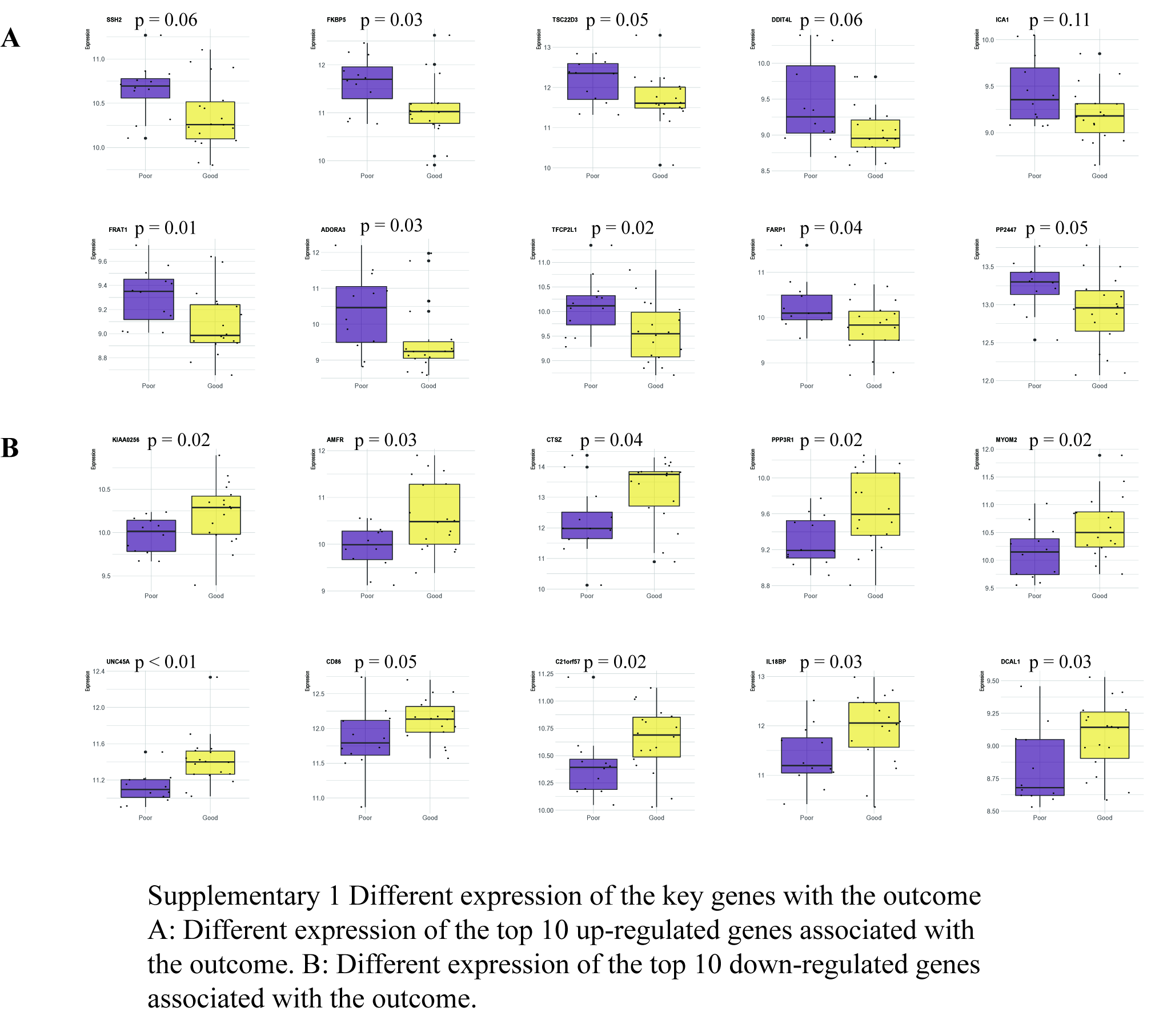

Supplement: Supplementary file 1 — Additional file 1. Supplementary 1 Different expression of the key genes with the outcome. [file 12890_2022_2130_MOESM1_ESM.tif]
